# Long-term tract-specific white matter microstructural changes after acute stress

**DOI:** 10.1007/s11682-020-00380-w

**Published:** 2020-09-11

**Authors:** Linghui Meng, Tong Shan, Kaiming Li, Qiyong Gong

**Affiliations:** 1 Huaxi MR Research Center (HMRRC), Department of Radiology, West China Hospital of Sichuan University, Chengdu, Sichuan China; 2 Department of Radiology, Third Hospital of Hebei Medical University, Shijiazhuang, Hebei China; 3 Institute of Public Health, Feinberg School of Medicine, Northwestern University, Chicago, IL USA; 4 Department of Radiology, Shengjing Hospital of China Medical University, Shenyang, Liaoning China; 5 Psychoradiology Research Unit of Chinese Academy of Medical Sciences, West China Hospital of Sichuan University, Chengdu, Sichuan China

**Keywords:** Acute stress, Trauma-exposed non-PTSD, DTI, Psychoradiology

## Abstract

Acute stress has substantial impact on white matter microstructure of people exposed to trauma. Its long-term consequence and how the brain changes from the stress remain unclear. In this study, we address this issue via diffusion tensor imaging (DTI). Twenty-two trauma-exposed individuals who did not meet post-traumatic stress disorder (PTSD) diagnostic criteria were recruited from the most affected area of Wenchuan earthquake and scanned twice (within twenty-five days and two years after the quake, respectively). Their emotional distress was evaluated with the Self-Rating Anxiety/Depression Scales (SAS/SDS) at both scans. Automatic fiber quantification was used to examine brain microstructure alterations. Correlation analyses were also conducted to investigate relationships between brain microstructure changes and symptom improvement. A group of demographically matched healthy controls (*N* = 22) from another project were scanned once before the quake using the same imaging protocols as used with trauma-exposed non-PTSD (TENP) participants. Two years after the earthquake, TENP individuals exhibited significantly reduced FA in the parietal portion of left superior longitudinal fasciculus and high FA in the parietal portion of left corticospinal tract. Over the follow-up, increased FA of the left uncinate fasciculus and the left corticospinal tract with parallel reduction of SAS and SDS were observed in TENP. No significant association was found between brain microstructure changes and symptom improvement. These results indicate changes in WM microstructure integrity of TENP brains parallel with symptom improvement over time after acute stress. However, the change would be a long-term process without external intervention.

## Introduction

Extreme traumatic events (i.e., natural disaster, accident, physical or sex abuse, combat) have significant adverse effects on more and more individuals around the world (Satcher et al. 2007), 46.9% of which are acute (Benjet et al. 2016). Less than 26% of those individuals will develop full-blown post-traumatic stress disorder (PTSD) (Qi et al. 2016). A large group of trauma-exposed people will not develop into PTSD but may still exhibit some signs of anxiety and depressive symptoms in the aftermath of the trauma. These trauma-exposed non-PTSD people (TENP) have showed different brain alterations compared with PTSD in prefrontal and limbic regions (Akiki et al. 2017; Li et al. 2014). Although structural and functional changes have been found in TENP shortly after trauma (Chen et al. 2013; Lui et al. 2013, 2009), the long-term brain alteration after acute stress remains largely unknown. Investigation of this matter may provide important insights on the brain’s mechanisms of resilience from acute stress.

White matter (WM) is a crucial neural substrate that connects distributed brain systems. Its microstructure can be affected both in short and long term after stress (McEwen and Gianaros 2011; McEwen and Morrison 2013). With diffusion tensor imaging (DTI), studies have found low WM fractional anisotropy (FA) after stress in both human and animals (Choi et al. 2009, 2012; Coplan et al. 2010; Lu et al. 2013) and linked the changes in FA with stress resilience and maladaptation (Admon et al. 2013; Galinowski et al. 2015). We have reported reduced FA in the prefrontal-limbic system in survivors shortly after a natural disaster (Chen et al. 2013). However, it is not clear how long the underlying WM microstructure deficits persist and how the brain changes over time in TENP.

In the DTI model, water diffusion can be characterized by the eigenvalues of the diffusion tensor (Mori and Zhang 2006). Based on three eigenvalues, FA is defined to characterize the anisotropy of the underlying water diffusion and it can measure the directionality of diffusion (Shi and Toga 2017). Additionally, radial diffusivity (RD, mainly a myelin marker reflecting diffusion perpendicular to axonal tracts) and axial diffusivity (AD, primarily an axonal marker which reflects diffusion along the principal direction of the fiber) are defined to describe the neurobiological determinants of altered white matter microstructure (Song et al. 2002, 2003, 2005). With these metrics, two major types of DTI analysis methods have been developed to investigate WM microstructure and structural connectivity. One is voxel-wise statistical analysis (e.g., tract-based spatial statistics (Smith et al. 2006)). It computes the diffusion properties of each voxel within the brain and performs voxel-wise statistical references accounting for multiple comparisons. However, this type of method might not have sufficient precision at an individual level due to the substantial morphological variability of fiber tracts among subjects (Wassermann et al. 2011). Tractography, another type of method for analyzing DTI data is widely used to identify the WM fascicles in the living human brain. It traces continuous fiber-tract trajectories along principal diffusion directions of local brain tissues, assuming that water diffuses faster along axons (Basser et al. 2000). Although it has superior tract correspondence across subjects, diffusion properties have been typically averaged over the entire tract. However, due to the variablity of diffusion metrics along a tract, averaging over the entire tract may obscure potentially important local alterations. Furthermore, comparing tract-based analysis with voxel-wise statistical studies is not always straightforward. It is difficult to relate the tract-based findings to voxel-wise clusters (Colby et al. 2012; Wang et al. 2017; Yeatman et al. 2012).

A recent advancement of tract-based analysis is automatic fiber quantification (AFQ), an algorithm that automatically identifies major white matter tracts in brain and compares measurements at anatomically equivalent locations along trajectories. AFQ allows researchers to compare diffusion metrics at multiple nodes along a tract and identify alterations locally. It has demonstrated significantly variable diffusion properties along tracts in both healthy people and those with psychiatric disorders (Hall et al. 2016; Sacchet et al. 2014; Wang et al. 2017; Yeatman et al. 2011), suggesting its superior sensitivity in examining WM microstructure integrity.

Previous studies lacked evidence regarding whether WM microstructure is abnormal along the entire tract or at specific locations on a tract in TENP after a relatively long period. We followed a group of TENP subjects, scanned them twice (within twenty-five days and two years after the quake, respectively), and employed AFQ to explore how WM microstructural characteristics change after a relatively long period in a cross-sectional design and confirm the affected tracts with a more sensitive longitudinal design. Considering our previous findings of decreased FA within 25 days after the traumatic event (Chen et al. 2013) and the persistence of abnormal functional connectivity in TENP after two years (Du et al. 2015), we hypothesized that: (i) compared with healthy controls (HC), TENP would exhibit alterations in WM tracts at follow-up; and (ii) compared with baseline, TENP would show improvement in microstructural integrity over the two-year follow-up, especially in WM tracts and specific locations implicated in stress response and emotion regulation.

## Methods

### Participants

22 healthy trauma survivors (age: 38.4 ± 11.2 years), were recruited from the most affected area of the Wenchuan earthquake and followed up for two years. They underwent the first MRI scan within 25 days (13–25 days) after the earthquake (baseline). Two years (686–821 days) after the earthquake, they were scanned a second time (follow-up). Before each MRI scan, a psychiatrist used a structured psychiatric interview (SCID-IV) to rule out a current or past psychiatric disorder (Spitzer et al. 1992). As a recent investigation has reported that the anxiety and depression symptoms were the survivors’ dominant reaction following the earthquake (Fan et al. 2011), we evaluated symptoms of emotional distress using the Self-Rating Anxiety/Depression Scales (SAS/SDS) which are standardized ratings to evaluate anxiety and depression symptoms. The higher the scores are, the severer of the symptoms (Zung 1965, 1971). Each participant spent about 2 hours in our laboratory for MRI examination and psychological analysis. At both scans, all the earthquake trauma survivors were free of mental and physical disorders and were not taking antidepressant or anxiolytic medication.

All TENP subjects were included if they: (1) physically experienced the earthquake and personally witnessed death, serious injury, and the collapse of buildings; (2) had no head injury, mental and physical disorder history, including PTSD; (3) experienced no significant traumatic events following the earthquake, or received antidepressant or anxiolytic drugs during the follow-up period; and (4) reported no serious traumatic events before the earthquake or history of psychiatric disorders in first degree relatives.

22 healthy controls (age: 38.6 ± 12.5 years), matched in age, sex and education, were recruited from Sichuan before the earthquake in another project. They performed the same procedures of MRI examination and psychological analysis as TENP subjects. They reported no significant lifetime history of traumatic events or psychiatric disorders in first degree relatives. HC subjects were only scanned once before the quake. The following exclusion criteria applied to both groups: neurological disorders, psychiatric disorders, alcohol or drug abuse, pregnancy, or any physical illness.

Since longitudinal HC data is unavailable, our primary analysis was to compare the microstructural characteristics between HC and TENP subjects in a cross-sectional design. Our secondary analysis was to confirm and localize in TENP the affected tracts with a more sensitive longitudinal design.

This study was approved by the Institutional Review Board of West China Hospital of Sichuan University. Each participant was provided written informed consent prior to the psychiatric evaluation and MRI session.

### Image acquisition

All DTI data were obtained on a GE EXCITE 3 T MR scanner with an 8-channel phase array head coil. A single-shot spin-echo echo planar image (SE-EPI) sequence with the following parameters was performed: repetition time/echo time (TR/TE) = 10,000/70.8 ms; matrix = 256 × 256; field of view (FOV) = 240 × 240 mm^2^; Flip angle (FA) = 90°; slice thickness = 3 mm no gap, 50 contiguous axial slices. The diffusion sensitizing gradients were applied along 15 non-collinear directions (b = 1000 s/mm^2^). An image without gradients (b0) was also acquired. Daily quality assurance, including a spin echo sequence to warm up the scanner and to verify the signal-to-noise ratio of images, was performed to ensure the quality and consistency of acquired images. Foam cushions were used to reduce head movement, and the acquired MR images were further inspected by an experienced neuroradiologist for possible head movement.

### DTI preprocessing

DTI data were preprocessed using the FDT toolbox of FSL (http://www.fmrib.ox.ac.uk/fsl) and VISTASOFT tools (http://white.stanford.edu/software/). The preprocessing procedure included the following steps: 1) eddy current correction with eddy_correct; 2) skull removal to create a brain mask with bet; 3) tensor model estimation using dtifit; and 4) conversion of nifti files to DT6 format as required by AFQ using VISTASOFT.

### Automatic fiber quantification

We used the AFQ toolbox (https://github.com/jyeatman/AFQ) for automatic identification and quantification of cerebral WM pathways (Yeatman et al. 2012). 20 major fiber tracts in an individual brain were identified. In order to identify localized alterations in diffusion imaging metrics along the tracts and corroborating results across studies (Colby et al. 2012; Wang et al. 2017; Yeatman et al. 2012), each fiber tract was evenly divided into 100 consecutive segments and each segment was represented by a node with the diffusion properties of the segment averaged and mapped to it. The vector of these consecutive nodes formed a TractProfile. Thus, besides mean diffusion parameters, AFQ can compute diffusion parameters by using a weighted sum of each fiber’s value at a given node, which helps improve the sensitivity for group comparison (Wang et al. 2017).

### Statistical analysis

Our primary analyses were to examine whether the TENP individuals still exhibited aberrant WM microstructure after two years and to characterize the WM alterations of the TENP individuals over the follow-up. All tests were two-tailed, with the alpha level set at 0.05. The SPSS software (Version 21.0, IBM, Armonk, NY; http://www.spss.com) was used for statistical tests of demographic and clinical information.

We have reported previously that trauma has an acute impact towards brain WM microstructure integrity (Chen et al. 2013). To examine whether the fiber integrity of TENP persisted over the two-year follow-up, FA values of the entire tracts and nodes along the tracts were compared between TENP and HC using two-sample t-test. To further confirm the fiber integrity alterations in above affected tracts, post-hoc analyses on FA of the nodes with significant changes were compared longitudinally by using paired t-test. To examine how the fiber integrity of TENP changed over the two-year follow-up, FA values of the entire tracts were compared in TENP longitudinally, post-hoc analyses on FA of the nodes with significant changes on entire tract were compared by using paired t-test. To examine the possible leading factor of FA abnormality, post-hoc comparisons of AD and RD were performed for the possible leading factor of aberrant FA values.

Given the possible correlation between neighboring segments of a tract, Bonferroni correction would be over conservative and inappropriate (Yeatman et al. 2012). Thus, we applied false discovery rate (FDR) (Genovese et al. 2002) correction to the total number of statistical tests, which was 100 (segments) × 20 (tracts) in the cross-sectional analysis and 100 segments per tract in the post-hoc longitudinal analysis. The alpha level was set at 0.05.

Exploratory correlation analyses were conducted to examine the possible association between DTI derived measures and SAS/SDS at the follow-up, and between changes of DTI derived measures and symptom improvement (SDS and SAS) over the follow-up.

## Results

### Comparison of demographic and clinical information

Age (t = −0.483, *p* = 0.632), sex (χ2 = 0.096, *p* = 0.757) and education (t = 0.462, *p* = 0.646) did not significantly differ between TENP individuals and control subjects (Table 1). Over two-year follow-up, TENP individuals exhibited significant symptom improvement as seen in the reduction of SAS (t = 3.440, *p* = 0.003) and SDS (t = 2.685, *p* = 0.015) (Table 1).

**Table 1:** Demographic and clinical information of participants

Characteristics	TENP (*n* = 22)		HC (*n* = 22)
	Baseline	Follow-up	
Age (years)	38.4 ± 11.2	40.4 ± 11.2	38.6 ± 12.5
Education (years)	8.3 ± 4.0	8.3 ± 4.0	8.9 ± 3.8
Female to male, n	8:14	8:14	9:13
Time after quake (days)	21.5 ± 3.7	732.2 ± 49.3	
SAS scores	46.2 ± 11.8	34.1 ± 10.4	
SDS scores	47.1 ± 11.2	37.5 ± 9.8	

*Abbreviations*: *SAS* self-rating anxiety scale, *SDS* self-rating depression scale, *TENP*, trauma-exposed non-PTSD. s.d., standard deviation. Values are presented in mean ± s.d. format

### Microstructure abnormality after two years (TENP_follow-up_ vs HC)

#### Mean WM measures over tracts

No group difference in mean FA was identified between TENP and HC for any of the 20 resultant fiber tracts from AFQ.

#### Node-wise comparison of TractProfile

**Fig. 1:**
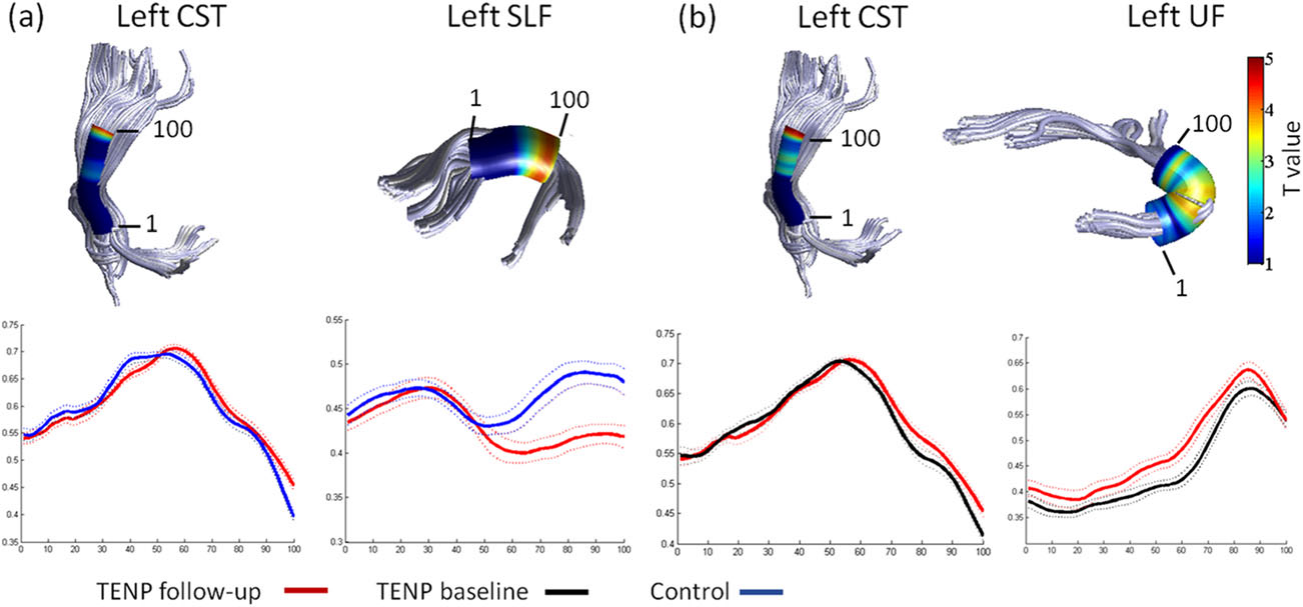
FA differences between TENP and healthy controls (**a**) and FA alterations over the follow-up in TENP (**b**). In each sub-figure, an example fiber tract is depicted in the upper panel with the starting and ending nodes marked with “1” and “100”, respectively; the corresponding TractProfile is shown in the lower panel, with y-axis standing for the FA value of each node and x-axis for all the 100 nodes along the tract. A solid line represents mean FA and a dotted line denotes standard error of FA. Abbreviations: TENP, trauma-exposed non-PTSD; CST, corticospinal tract; SLF, superior longitudinal fasciculus; UF, uncinate fasciculus

**Fig. 2:**
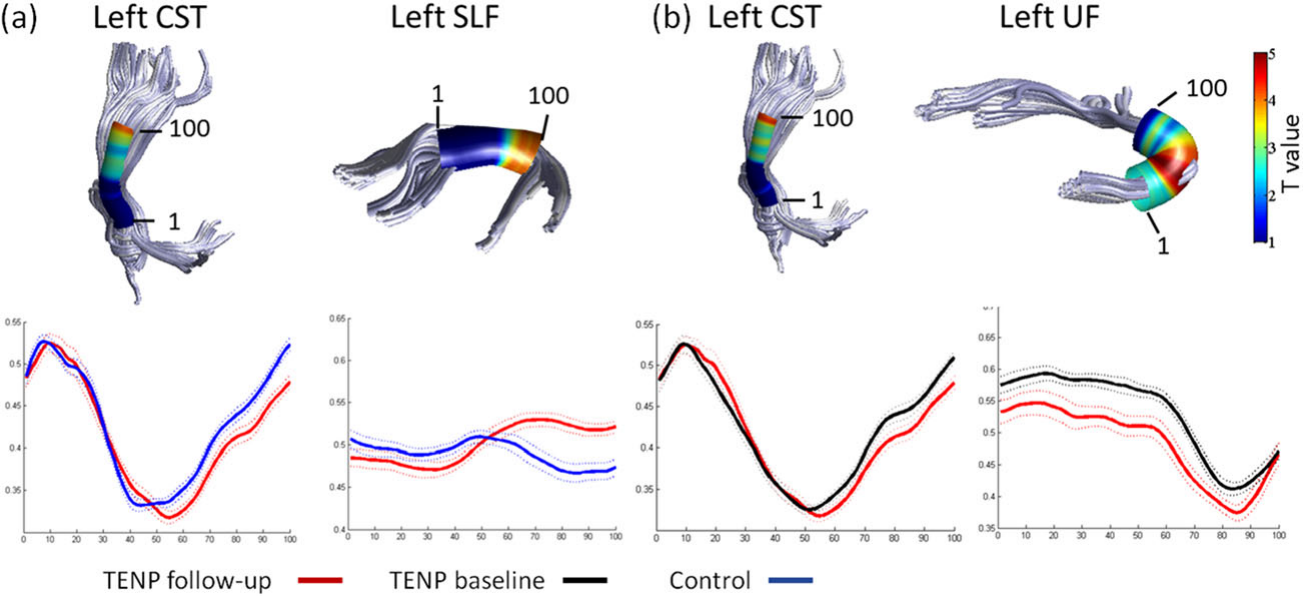
RD differences in nodes with significant FA differences. Similarly, as in Fig. 1, an example fiber tract is depicted in the upper panel and the corresponding TractProfile is shown in the lower panel. A solid line represents mean RD and a dotted line denotes standard error of RD. Abbreviations: TENP, trauma-exposed non-PTSD; CST, corticospinal tract; SLF, superior longitudinal fasciculus; UF, uncinate fasciculus

For the left corticospinal tract (CST), the TENP individuals showed significantly higher FA (t: 3.858 ~ 4.422, p: 0.026 ~ 0.030, Cohen’s d: 1.163 ~ 1.333) (Fig. 1a) and lower RD (t: −4.107 ~ −4.056, *p* = 0.013, Cohen’s d: 1.223 ~ 1.238) (Fig. 2a) in nodes 98–100, compared with HC. For the left superior longitudinal fasciculus (SLF), TENP had significantly lower FA (t: −4.186 ~ −3.628, p: 0.026 ~ 0.048, Cohen’s d: 1.094 ~ 1.262) (Fig. 1a) and higher RD (t: 3.841 ~ 4.068, p = 0.013, Cohen’s d: 1.158 ~ 1.227) (Fig. 2a) in nodes 76–97 compared with the control group. None of above nodes showed significant AD changes. No significant correlation was found between FA and SAS/SDS or between RD and SAS/SDS.

### Longitudinal alterations over the follow-up in TENP (TENP_follow-up_ vs TENP_baseline_)

#### Mean WM measures over tracts

In the TENP group, mean FA values were increased over the follow-up in the left uncinate fasciculus (UF) (t = 4.277, *p* = 0.007, Cohen’s d: 0.912) (Fig. 1b), accompanied with reduced RD (t = −4.789, *p* = 0.002, Cohen’s d: 1.021) (Fig. 2b). There was no significant AD change in the tract.

#### Node-wise comparison of TractProfile

In TENP individuals, nodes 98–100 of the left CST and 31–72, 82–90 of the left UF demonstrated increased FA (shown in Fig. 1b for CST, t: 4.671 ~ 4.866, *p* = 0.004, Cohen’s d: 0.996 ~ 1.037; for UF, t: 2.439 ~ 3.731, p = 0.013 ~ 0.047, Cohen’s d: 0.520 ~ 0.795) and decreased RD (shown in Fig. 2b for CST t: −4.211 ~ −4.055, *p* = 0.009, Cohen’s d: 0.898 ~ 0.865; for UF, t: −5.123 ~ −2.424, *p* = 0.001 ~ 0.032, Cohen’s d: 1.092 ~ 0.517). Neither tract showed significant AD changes in any node. No significant association was found between changes of FA/RD and SAS/SDS.

## Discussion

The present study followed up a group of TENP individuals for two years. We examined both cross-sectional tract-specific WM microstructure abnormalities of TENP compared with HC and longitudinal tract-specific WM alterations in TENP individuals over the follow-up. Significantly lower FA in the left SLF and higher FA in the left CST were observed in the parietal area of TENP compared with HC after two years of the earthquake. Longitudinally, increased FA of the left UF and CST with a parallel reduction of emotional symptoms were observed in TENP individuals over the follow-up.

With the same group of trauma exposed individuals at baseline, we have previously reported an acute impact of the earthquake (within 25 days) on human brain microstructure, i.e., reduced cerebral microstructure within the prefrontal-limbic system and parietal lobe (Chen et al. 2013). After two years, TENP individuals still demonstrated aberrant FA values in the parietal segments of SLF and CST compared with HC, indicating that the affected WM microstructure integrity in TENP persisted after two years. This is consistent with previous studies showing that trauma exposure has a long-term impact on the brain structure and function (Du et al. 2015; Ganzel et al. 2008). Importantly, the TractProfile analyses revealed tract-specific alterations in TENP. Lower FA in the left SLF and higher FA in the left CST were observed in the parietal lobe of TENP after two years of the earthquake. While the alterations in the parietal area appeared localized, the increase of FA in UF were relatively diffused. Particularly, FA increase of the left UF and CST paralleled reduction of emotional symptoms in TENP individuals over the follow-up, suggesting that these tracts and associated cortices may play important role in the brain’s resilience from acute stress.

Reduced FA at the parietal portion of SLF has been associated with depression and PTSD studies (Hu et al. 2016; Zou et al. 2008), indicating that this region has a crucial role in mood regulations. Whether the FA reduction is resulted from or leading to functional deficit in the corresponding cortex warrants further investigation. Our previous study also has showed reduced FA in the parietal lobe of trauma exposed individuals within a short time after trauma (Chen et al. 2013). The current study demonstrated that the WM microstructure integrity in parietal regions of TENP still exhibited abnormality compared with HC after two years (p: 0.026 ~ 0.048), and no integrity improvement for the tract was found longitudinally over the two years (p: 0.864 ~ 0.993). Considering that the stress symptoms have been much alleviated over the follow-up, the lower WM microstructure integrity in parietal portion of SLF might be more of a major risk factor predisposing patients to development of PTSD rather than an adaptive response to acute stress from the trauma. This warrants further studies with a larger sample size.

CST showed higher FA in the parietal segments comprising corona radiata. Traditionally, CST is considered to play a primary role in voluntary movement control. Recent studies, however, have shown that CST also projects from the somatosensory, cingulate, and insular cortices (Galea and Darian-Smith 1994; Nii et al. 1996; Fukuda et al. 2008; Kumar et al. 2009). Our result provides additional evidences that CST may be involved in affective processing (Moreno-Lopez et al. 2016; Sacchet et al. 2014).

Furthermore, the regions with higher FA in CST and lower FA in SLF both locate in parietal portions. Previous research has reported that FA increase in the CST is associated with FA reduction in SLF at the similar region in Alzheimer’s disease (Douaud et al. 2011). Considering that FA reduction in corona radiate and CST has been associated with PTSD and depressive disorder (Guo et al. 2012; Hu et al. 2016; Schuff et al. 2011) and that the FA of CST has increased during the two-year follow-up (*p* = 0.004), we speculate that the increase of WM microstructure integrity at the parietal portion of CST may be a compensatory response to aberrant WM microstructure integrity in SLF at the same region in TENP after acute stress. However, it should be noted that no significant correlation was found between FA increase in CST and symptom alleviation. This might be related to the small sample size, which limits the power of our analyses. Longitudinal DTI studies on PTSD are necessary to confirm this hypothesis.

FA abnormalities in the prefrontal-limbic system, which had been found shortly after the quake (Chen et al. 2013), disappeared in TENP after two years, compared with HC. Further, accompanying stress symptom improvement, a longitudinal comparison of TENP over the follow-up revealed statistically increased FA in UF, a primary associative fiber tract that anatomically connects parts of the prefrontal-limbic system (Admon et al. 2013; Schmahmann et al. 2007; Wang et al. 2017). This is consistent with previous study which showed reduction in structural integrity of UF being related to maladaptive responses to stress (Admon et al. 2013). These results clearly showed that a reversible process of WM integrity in the prefrontal-limbic system paralleling with the stress symptom improvement in TENP. This suggests that the underlying WM structure of the system is plastic to both trauma and resilience in healthy survivors.

Unlike CST and SLF, UF showed diffused FA increase along the entire tract, especially at the trunk of UF. This result may be relevant to extensive functional change and increased functional connectivity in the corresponding cortices, i.e., vmPFC and hippocampus (Admon et al. 2009). However, no significant association was found between FA and SAS/SDS. Future diffusion studies with finer resolution are necessary for further assessment of relationship between FAs of UF and symptom improvement in TENP.

Our analysis revealed aberrant FA in WM structures, accompanied by aberrant RD rather than AD. FA, RD, and AD are widely used DTI metrics to characterize the diffusion properties of WM microstructure. FA is an aggregate index of WM integrity, which reflects multiple factors of WM, including myelination, fiber orientation, intra/extra-cellular volume and packing density of WM fibers (Chan et al. 2010; Li et al. 2016). AD is primarily an axonal marker which reflects diffusion along the principal direction of fibers, while RD is mainly a myelin marker reflecting diffusion perpendicular to axonal tracts (Song et al. 2002, 2003, 2005). Animal studies have shown that increased RD is associated with reduced myelination (Harsan et al. 2007), whereas reduced RD is related to remyelination (Song et al. 2005). Furthermore, demyelination by stress has also been shown in animal models (Hemanth Kumar et al. 2014). Our results suggested that the microstructure integrity changes in TENP may be primarily a result of myelin changes, and myelin in the human brain may be adversely affected by severe stress.

Several issues need to be considered when interpreting our results. First, the sample size in this study is relatively small and we did not use standard scales to measure individual trauma ratings. Second, a limitation of AFQ is that only a central portion of the fiber tract is analyzed. Third, our DTI imaging protocol with an 8-channel head coil and a low number of gradient directions may exclude more sophisticated diffusion modeling. This could be alleviated by multi-band and multi-shell diffusion imaging techniques in the future. Fourth, some confounding factors, including childhood/early stress and scan environmental changes, cannot be excluded in the analysis. Finally, the control individuals were scanned only once, and we are unable to model the group by time effects via an ideal repeated-measure longitudinal analysis.

## Conclusions

Our two-year follow-up of a group of TENP individuals demonstrated changed WM microstructure integrity of TENP brains paralleled with symptom improvement over time after acute stress. However, the change would be a long-term process without external intervention.
